# Electronically Coupled Heterojunctions Based on Graphene and Cu2−xS Nanocrystals: The Effect of the Surface Ligand

**DOI:** 10.3390/molecules30010067

**Published:** 2024-12-27

**Authors:** Ju Y. Shang, Mariangela Giancaspro, Adriana Grandolfo, Rafique A. Lakho, Elisabetta Fanizza, Suraj K. Patel, Giuseppe Valerio Bianco, Marinella Striccoli, Chiara Ingrosso, Oscar Vazquez-Mena, M. Lucia Curri

**Affiliations:** 1 Program of Materials Science, University of California San Diego, La Jolla, CA 92093, USA; y1shang@ucsd.edu (J.Y.S.); sup027@ucsd.edu (S.K.P.); 2 Aiiso Yufeng Li Family Department of Chemical and Nano Engineering, Center for Memory and Recording Research, University of California San Diego, 9500 Gilman Drive #0401, La Jolla, CA 92093, USA; 3 Department of Chemistry, University of Bari, Via Orabona 4, 70126 Bari, Italy; mariangela.giancaspro@uniba.it (M.G.); adriana.grandolfo@poliba.it (A.G.); rafique.ahmed@poliba.it (R.A.L.); elisabetta.fanizza@uniba.it (E.F.); marialucia.curri@uniba.it (M.L.C.); 4 National Research Council of Italy—Institute for Physical and Chemical Processes (CNR-IPCF), Bari Division, Via Orabona 4, 70126 Bari, Italy; m.striccoli@ba.ipcf.cnr.it; 5 Department of Electrical and Information Engineering, Polytechnic of Bari, Via Amendola 126/b, 70126 Bari, Italy; 6 Department of Aerospace Science and Engineering, Polytechnic of Bari, Via Amendola 126/b, 70126 Bari, Italy; 7 CNR-NANOTEC c/o Department of Chemistry, University of Bari, Via Orabona 4, 70126 Bari, Italy; giuseppevalerio.bianco@cnr.it

**Keywords:** graphene, nanocrystals, ligand, heterojunction, photodetector, responsivity

## Abstract

Optoelectronic devices combining single-layer graphene (SLG) and colloidal semiconducting nanocrystal (NC) heterojunctions have recently gained significant attention as efficient hybrid photodetectors. While most research has concentrated on systems using heavy metal-based semiconductor NCs, there is a need for further exploration of environmentally friendly nanomaterials, such as Cu_2−x_S. Chemical ligands play a crucial role in these hybrid photodetectors, as they enable charge transfer between the NCs and SLG. This study investigates the photoresponse of an SLG/Cu_2−x_S NCs heterojunction, comparing the effect of two short molecules—tetrabutylammonium iodide (TBAI) and 3,4-dimethylbenzenethiol (DMBT)—as surface ligands on the resulting structures. We have analysed charge transfer at the heterojunctions between SLG and the Cu_2−x_S NCs before and after modification with TBAI and DMBT using Raman spectroscopy and transconductance measurements under thermal equilibrium. The photoresponse of two hybrid devices based on three layers of Cu_2_₋_x_S NCs, deposited in one case on SLG/Cu_2−x_S/TBAI (“TBAI-only” device) and in the other on SLG/Cu_2−x_S/DMBT (“DMBT + TBAI” device), with a TBAI treatment applied, for both, after each layer deposition, has been evaluated under 450 nm laser diode illumination. The results indicate that the TBAI-only device exhibited a significant increase in photocurrent (4 μA), with high responsivity (40 mA/W) and fast response times (<1 s), while the DMBT + TBAI device had lower photocurrent (0.2 μA) and responsivity (2.4 μA), despite similar response speeds. The difference is attributed to DMBT’s π–π interactions with SLG, which enhances electronic coupling but reduces SLG’s mobility and responsivity.

## 1. Introduction

Heterojunctions formed of graphene and narrow band gap inorganic colloidal semiconductor nanocrystals (NCs) have shown promising attributes and performance for field effect transistors (FETs), photodiodes, solar cells, and photodetectors. As a charge collector and transport layer, graphene offers wide bandwidth, high charge mobility, fast response time, charge density tunability by electrostatic gating, and charge transfers from adjacent species, in addition to a high Young’s modulus beneficial for flexible devices [1]. As light absorbers, semiconducting NCs present high exciton generation capability due to their direct band-gap, strong size-tuneable light harvesting properties, and spectral selectivity [2]. Under thermal equilibrium conditions, the difference in work function between the semiconducting NCs and graphene induces charge transfers, leading to the alignment of their Fermi levels and establishing a built-in electric field at the heterojunction [3]. Under light illumination, the built-in potential drives a net charge transfer to graphene, resulting in a change of its carrier density, generating a photoresponse dependent on the spectral absorption of the NCs [3].

Colloidal NCs of diverse chemical composition and light absorption in different spectral ranges have been used to manufacture photoactive heterojunctions with graphene [4,5,6,7] Among these, heterojunctions of multilayer intercalating stacks of PbS NCs and single-layer graphene (SLG) implemented in hybrid solar cells, have shown a high power conversion efficiency of 9.18% and current density of 24.09 ± 0.46 mA/cm^2^, due to an efficient extraction and transportation of photogenerated charges and a large interface area between the NCs and the graphene interlayers [5]. SLG modified by CdSe/CdS/ZnS NCs films has been used in light-emitting diodes, showing current and power efficiencies higher than similar devices based on ITO under low current density [6]. SLG coupled with colloidal PbS NCs films have been integrated into photodetectors, where the fast transit time of the charges to graphene and the long lifetime of carriers in the NCs induce a large gain mechanism, producing multiple charge carriers in the SLG channel from one incident photon, which result in a responsivity (10^9^ A/W) and a photodetection gain enhanced with respect to the bare SLG-based counterpart at an extremely low incident illumination power (below 10^−14^ W) [7]. Despite their excellent performance, the use of heavy metal-based semiconductor NCs, such as those containing cadmium or lead, poses significant risks to human health and the environment. Consequently, recent research has shifted towards exploring nanostructured semiconductors made from alternative elements, including copper, indium, sulphur, and phosphine [8,9,10].

To the best of our knowledge, few studies have reported on the characterisation of such heterojunctions for optoelectronics. Specifically, a photodetector integrating a hybrid nanocomposite formed of reduced graphene oxide and Cu_2_S NCs, synthesised in situ on GO sheets using oleylamine (Olam) as a coordinating ligand and reducing agent, has shown a photocurrent generation of 1.25 nA under the illumination of a 405 nm laser at 30 mW power [11].

**Figure 1:**
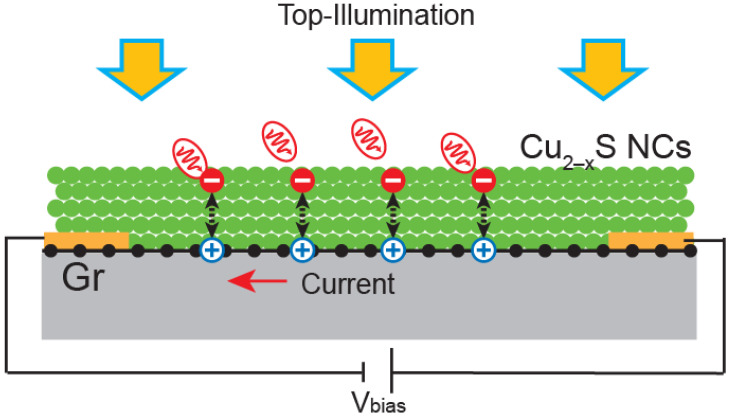
Hybrid photodetector based on SLG, coated with a film of Cu_2−x_S NCs. The device is built on top of a SiO_2_/Si chip with gold contacts to apply voltage on the SLG. The device is illuminated from the top. The NCs absorb light and generate photocarriers that are transferred to SLG, increasing the number of carriers in SLG and inducing an increase in conductance.

Herein, we report on the fabrication and characterisation of hybrid photodetectors integrating heterojunctions based on SLG, grown by chemical vapour deposition, coated with Cu_2−x_S NCs synthesised by a colloidal approach, as shown in Figure 1. One advantage of this configuration is that it benefits from the high mobility of graphene and the strong light absorption and photocarrier generation in NCs. The photocarriers in the NCs are transferred to SLG due to the built-in potential at the interface, leading to a significant change in the current in SLG. In particular, the large mobility of graphene leads to a short transit time in SLG (τ_transit_), which, combined with a long photocarrier lifetime in the NCs (τ_lifetime_), yields a strong photogain (G = τ_lifetime_/τ_transit_) and high responsivity compared to pure NCs photodetectors [3,12]. It is important to stress that this is not a composite material based on mixing the two materials, but rather a single heterojunction using a thin film of Cu_2−x_S NCs and a high-mobility SLG. Cu_2−x_S NCs have been selected for their tuneable stoichiometry, which allows for adjustable optical and electronic properties, making them highly suitable for photodetection applications. These NCs exhibit broadband light absorption across the UV-Vis-NIR spectral range and display a self-doped p-type semiconductor behaviour due to copper vacancies, enhancing photodetection performance [12,13].

In this study, we have focused on the modification of the surface ligands of the Cu_2−x_S NCs, originally coated with insulating ligands such as oleylamine and oleic acid (Olam/OA), to enhance charge transfer to SLG. Indeed, ligands like Olam and OA are widely used in the colloidal synthesis of NCs due to their effectiveness in controlling nucleation and growth [11], and to obtain stable dispersion in solvents.

However, due to their long alkyl chain, they act as dielectric layers hampering charge transfer both between NCs and between NCs and SLG, significantly reducing the photoresponse of the heterojunction. To address this, replacement of the long-chain ligands with shorter molecules (i.e., tetrabutylammonium iodide (TBAI), 1,2-ethanedithiol (EDT), ethanolamine and butylamine etc.) has been demonstrated to promote charge transfer to SLG, passivate trap sites on NC surfaces to limit charge recombination, and extend the lifetime of carriers [14,15]. Studies have shown that electron conductivity within NC-deposited films increases exponentially as ligand chain length decreases, facilitating efficient charge collection in SLG [16]. Additionally, treatments with shorter ligands can modify the valence and conduction band energy levels of the NCs [17], adjusting their band offset with the Fermi level of SLG, thus improving NCs charge injection. For example, photodetectors featuring heterojunctions of SLG modified with Olam-coated Cu_2−x_Se NCs and treated with butylamine have demonstrated an enhanced photogating effect. These devices produced a photocurrent of approximately 48 µA and exhibited high photoresponsivity when illuminated with a 405 nm laser diode at an intensity of 2.5 mW/cm^2^ [18]. Moreover, it has been shown that using aromatic molecules as surface ligands of the NCs at the interface with SLG in photodetectors can significantly enhance photoresponsivity and charge collection. This improvement has been ascribed to aromatic π–π stacking interactions between the ligands and SLG, which strengthen charge coupling between SLG and NCs. For instance, photodetectors with SLG films modified by 1-pyrene butyric acid-coated PbS NCs have shown an increase of 30% of the photocurrents with respect to analogous devices integrating heterojunctions formed of TBAI-coated PbS NCs [19]. Photodetectors based on SLG films, modified by benzendithiol-coated Cu_3−x_P NCs and having a broadband photoresponse from 400 to 1550 nm, have shown an ultrahigh responsivity and a high photoconductive gain under the illumination of a diode laser at 405 nm [8].

In this work, we have studied and compared the effect of two short ligands, namely the aromatic 3,4-dimethylbenzenethiol (DMBT) and the ionic tetrabutylammonium iodide (TBAI) at the interface between SLG and Cu_2−x_S NC layer on the photoresponse of hybrid photodetectors. We have analysed charge transfer at the heterojunctions between SLG and the Cu_2−x_S NCs, before and after modification with TBAI (SLG/Cu_2−x_S/TBAI) and DMBT (SLG/Cu_2−x_S/DMBT), respectively, as surface ligands, using Raman spectroscopy and transconductance measurements under thermal equilibrium condition. The photoresponse of two hybrid devices based on three layers of Cu_2_₋_x_S NCs deposited in one case on SLG/Cu_2−x_S/TBAI (“TBAI-only” device) and in the other on SLG/Cu_2−x_S/DMBT (“DMBT + TBAI” device), with a TBAI treatment applied, in both cases, after each layer deposition, has been evaluated under 450 nm laser diode illumination. The results indicate that the TBAI-only device exhibited a significant increase in photocurrent (4 μA), with high responsivity (40 mA/W) and fast response times (<1 s). In contrast, the DMBT + TBAI device, while maintaining fast response times, demonstrates a lower photocurrent (0.2 μA) and a reduced responsivity (2.4 μA/W). Overall, the TBAI-only device outperforms the DMBT + TBAI device in terms of photocurrent and responsivity despite both showing fast response times. This difference has been explained considering the aromatic π–π stacking interactions between DMBT and the SLG basal plane in the SLG/Cu_2−x_S/DMBT heterojunction, which, while improving electronic coupling and shifting the Fermi level more than in the TBAI-only configuration, as indicated by transconductance measurement, ultimately reduce SLG’s mobility and decrease conductance and overall responsivity.

## 2. Results and Discussion

**Figure 2:**
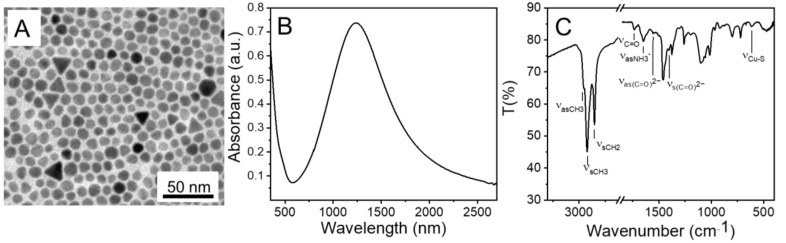
(**A**) TEM image, (**B**) UV-Vis-NIR absorption spectrum and (**C**) ATR-FTIR spectrum of Olam/OA-capped Cu_2−x_S NCs.

Olam/OA-capped Cu_2−x_S NCs were synthesised by means of the hot injection method reported in M. Giancaspro et al. [20]. A two-pots strategy was used, consisting of the thermal decomposition of the S_8_ precursor in an OA and octadecene (ODE) mixture, followed by its rapid injection into a CuCl_2_ precursor solution in a mixture of the coordinating ligands OA and Olam, in ODE, at 200 °C, for allowing NCs nucleation followed by growth at 180 °C. The synthesised Olam/OA-Cu_2−x_S NCs have relatively good monodispersion and show a spherical shape with an average diameter of 9 nm, with a percentage of polydispersity of 6% (Figure 2A). The UV-Vis-NIR absorption spectrum of the NCs shows a wide signal at wavelengths higher than 500 nm, which is centred at 1234 nm and corresponds to the Localised Surface Plasmon Resonance (LSPR) band originating from plasmon oscillations of free holes. The last is generated by copper vacancies in the valence band of the NCs, which are in the digenite Cu_1.8_S crystalline phase [20]. Besides, an intense absorption signal below 500 nm can be observed in the spectrum of the NCs, which is accounted for by their semiconductor behaviour (Figure 2B). The ATR-FTIR spectra of the Olam/OA-Cu_2−x_S NCs show both asymmetric (ν_as_) and symmetric (ν_s_) stretching vibrations of -CH_3_ groups, along with symmetric stretching (ν_s_) of -CH_2_ groups, which are attributed to Olam and OA (Figure 2C). The characteristic stretching vibration mode of Cu-S is evident in the 550–650 cm^−1^ wavenumber range (Figure 2C) [21]. A peak at 1734 cm^−1^ corresponds to the stretching vibration (ν_s_) of the -C = O groups of OA, indicating a monodentate binding to the NC surface. Additionally, two bands around 1550 cm^−1^ and 1403 cm^−1^ can be observed (Figure 2C), associated with the asymmetric (ν_as(C = O)2−_) and symmetric stretching (ν_s(C = O)2−_) of the carboxylate groups from OA, which partially coordinate the NP surface through a bidentate bonding [20]. These spectral features support the idea that both carbonyl and carboxylate groups are involved in binding to the surface of the prepared NCs. Finally, the spectrum presents a peak at 1648 cm^−1^ (Figure 2C) ascribed to the asymmetric stretching of -NH_3_^+^ groups (ν_asNH3+_) reflecting the acid–base equilibrium between Olam and OA ligands in the formation of the Olam^+^ OA^−^ complex [20].

**Figure 3:**
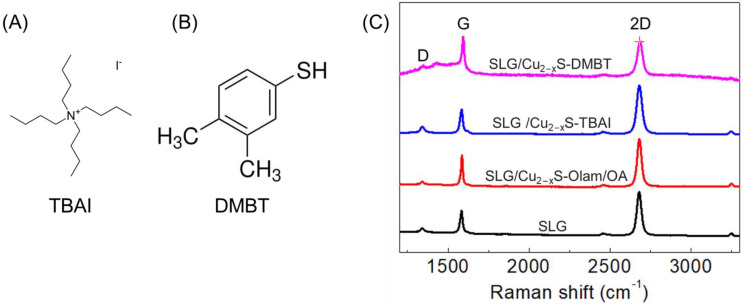
(**A**) Chemical structure of tetrabutylammonium iodide (TBAI) and (**B**) 3,4-dimethylbenzenethiol (DMBT). (**C**) Raman spectra of SLG (black trace), SLG modified with the as-synthesised Olam/OA-Cu_2−x_S NCs (red trace: Cu_2−x_S), and after treatment with TBAI (blue trace: SLG/Cu_2−x_S/TBAI) and DMBT (pink trace: SLG/Cu_2−x_S/DMBT).

Raman spectroscopy was performed to investigate the structural properties of SLG before and after modification with the NCs and after treatment with TBAI and DMBT. The Olam/OA-Cu_2−x_S NCs were spin-coated onto SLG, followed by treatment with the tetrabutylammonium iodide (TBAI) anionic ligand and the 3,4-dimethylbenzenethiol (DMBT) aromatic ligand, respectively (Figure 3A,B). Bare SLG shows two strong signals at 1584 and 2679 cm^−1^, which are, respectively, ascribed to the G and 2D peaks of the graphitic platform, along with a weak signal at 1338 cm^−1^ due to the D peak (Figure 3C). The Raman-forbidden D and D′ bands are also visible in the spectrum as, respectively, a single peak at 1338 cm^−1^ and a shoulder of G peak around 1620 cm^−1^. They need a structural defect to be activated to satisfy the momentum conservation in the Raman scattering process. CVD graphene typically presents intrinsically structural defects such as C-sp^3^ atoms, vacancies and extended line defects at grain boundaries due to its polycrystalline nature. While the D peak relative intensity is used as a measure of the material structural order in monolayer graphene [22], the intensity ratio between the D and D′ peak depends on the nature of the defects in the carbon lattice area irradiated by the laser beam during Raman measurements. Specifically, a lower D/D’ intensity ratio occurs when the laser beam is in proximity to graphene grain boundaries [22]. After deposition of the Olam/OA-Cu_2−x_S NCs, the G and 2D peaks are almost at the same wavenumbers, namely at 1582 and 2671 cm^−1^, respectively, and they maintain almost the same position also after treatment with TBAI, staying at 1585 and 2673 cm^−1^ (Figure 3C). Also, the D/G ratio keeps almost the same intensity, attesting to the preservation of the conjugated sp^2^ carbon lattice of SLG after deposition of the NCs and treatment with TBAI. After treatment with DMBT, the Raman signal of the aromatic thiol affects the spectrum baseline between D and G peaks. Moreover, the G and 2D peaks shift to higher wavenumbers, passing to 1593 cm^−1^ and 2684 cm^−1^, respectively, and their 2D/G ratio shows a significant decrease, passing from 1.7 to 0.97 (Figure 3C). This evidence indicates a change of the graphene electronic state, which is induced by an increase in the hole carrier concentration originating from transfers of the photogenerated holes from the NCs to SLG upon irradiation with the laser excitation source of 532 nm used in the Raman measurements, where the NCs show slight absorption (Figure 3B) [23].

**Figure 4:**
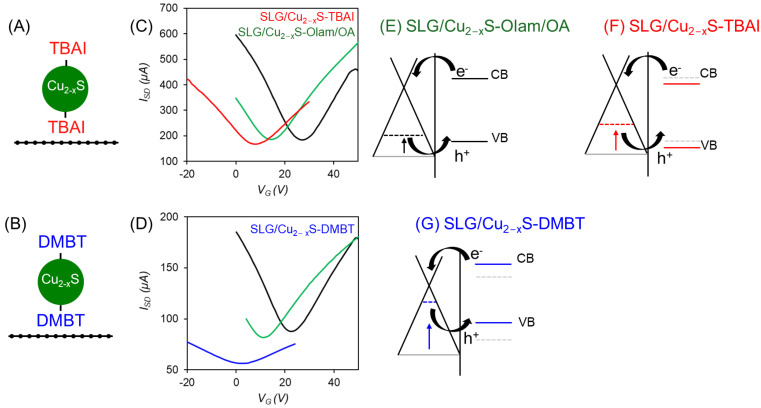
(**A**,**B**) Sketches of the SLG/Cu_2−x_S heterojunctions with a single spin-coated layer of NCs treated by TBAI and DMBT. (**C**,**D**) Transconductance measurements (I_SD_ vs. V_G_, V_DS_ =0.04 V) of bare SLG (black curve), SLG after deposition of Olam/OA-Cu_2−x_S NCs (green curve), and after TBAI (red curve) and DMBT (blue curve) treatment, showing the shift in V_DP_. TBAI induces a negative shift to V_DP_ = 8 V, while DMBT induces a larger shift to V_DP_ = 2 V. (**E**,**G**) Proposed band diagrams showing the shifts in Fermi level (E_F_) of SLG before and after ligand treatment. Deposition of the Cu_2−x_NCs before (**E**) and after treatment with TBAI (**F**) and DMBT (**G**) induces an upper shift of E_F_ towards the Dirac point but keeping SLG in a p-doped hole conduction state (below Dirac point).

Transconductance measurements were performed under dark and thermal equilibrium conditions to investigate the effect of TBAI and DMBT on the charge transfer between NCs and SLG. Two samples were prepared by spin-coating a single layer of Olam/OA-Cu_2−x_S NCs on SLG, which then were treated, respectively, by spin-coating TBAI and DMBT (Figure 4A,B). The bulk of the silicon chips was used as a back gate using the 300 nm silicon oxide as a gate dielectric with a source-drain voltage (V_DS_) of 0.04 V. The SLG-NC samples with one layer of NCs, respectively, functionalised with TBAI and DMBT ligands, are represented schematically in Figure 4A,B. The measured thickness of one layer of deposited NCs is approximately 35 nm. Figure 4C shows the transconductance curve of the neat SLG (black trace) after transfer onto the SiO_2_ substrate, with a negative slope for V_G_ = 0. This accounts for the p-type doping state of SLG, which is due to oxygen terminations at the SLG basal plane defects and grain boundaries, to interface effects with the substrate, and to physiosorbed contaminants from the air, as O_2_, H_2_O, and CO_2_ [5]. The Dirac point, which is typically observed as a minimum in the current versus V_G_ plot and labelled as V_DP_, is located at 27 V [19]. After spin-coating the Olam/OA-Cu_2−x_S NCs onto SLG, the transconductance trace (green trace) of the SLG/Cu_2−x_S sample, collected in thermal equilibrium conditions, is shifted to lower V_G_, reaching the Dirac point V_DP_ of 14 V. This evidence indicates an increase in the Fermi level (E_F_) of SLG, equilibrating with that of the NCs, which is induced by electron transfers from the NCs to SLG (Figure 4E). This transfer of charge is driven by the built-in potential generated at the heterojunction because of the work functions offset between the two materials, having SLG the E_F_ at ca. −4.6 eV [24] and the Cu_2−x_S NCs at ca. −5.79 eV [25]. After treatment with TBAI, which can replace the original capping layer [19], the transconductance curve (red trace) shows a further shift of E_F_ for SLG towards lower V_G_ (red trace in Figure 4C) with V_DP_ = 8 V (−6 V shift), indicating that E_F_ moves further upwards towards the Dirac point, but remaining below it (Figure 4F). This evidence can be accounted for by the coordination of halide ions (I^−^) that, displacing Olam/OA, behave as an electron-donating ligand [26,27], causing a shift of the VB of the NCs towards lower energies and modifying their band offset with the SLG’s E_F_ [26,27], favouring electron transfers to SLG (Figure 4F) and resulting in a p-type hole conduction. The transconductance measurements also allow us to extract the mobility of SLG after coating with NCs and TBAI treatment, obtaining a value of 4200 cm^2^/Vs and highlighting the high mobility of our SLG films. In the case of the DMBT sample, we observe a similar behaviour on the transconductance of SLG, as expected. For SLG with V_DP_ = 22 V, a shift down to V_DP_ = 12 V after Olam/OA-Cu_2−x_S NC coating is observed (Figure 4D). After ligand exchange with DMBT, a shift of E_F_ for SLG towards lower V_G_ = 2V is observed, indicating a greater difference in V_DP_ of −10V (blue trace in Figure 4D), and hence assessing a larger net transfer of electrons from DMBT-functionalised NCs to SLG (Figure 4G). This result is likely due to the electron-accepting nature of the thiophenol ligand DMBT, substituted with two electron-donating -CH_3_ groups, which increases the energy of the VB of the Cu_2−x_S NCs [28] and promotes a stronger built-in potential. The extent of the observed shift of E_F_ suggests the establishment of stronger coupling interactions between the Cu_2−x_S NCs and SLG that can be ascribed to DMBT-mediated aromatic π–π stacking interactions between the SLG basal plane and the NCs [25]. However, the blue trace for the SLG/Cu_2−x_S/DMBT heterojunctions in Figure 4D shows a marked reduction in both current and current-gate voltage slope compared to the red trace for the SLG/Cu_2−x_S/TBAI ones in Figure 4C. For the SLG coated with NCs with DMBT treatment, the mobility is 530 cm^2^/Vs, showing a significant reduction compared to the TBAI treatment. This suggests that the stronger aromatic interactions in the SLG/Cu_2−x_S/DMBT also result in a significant drop in SLG mobility, likely due to the alteration of the graphene band structure by the aromatic coupling [29].

**Figure 5:**
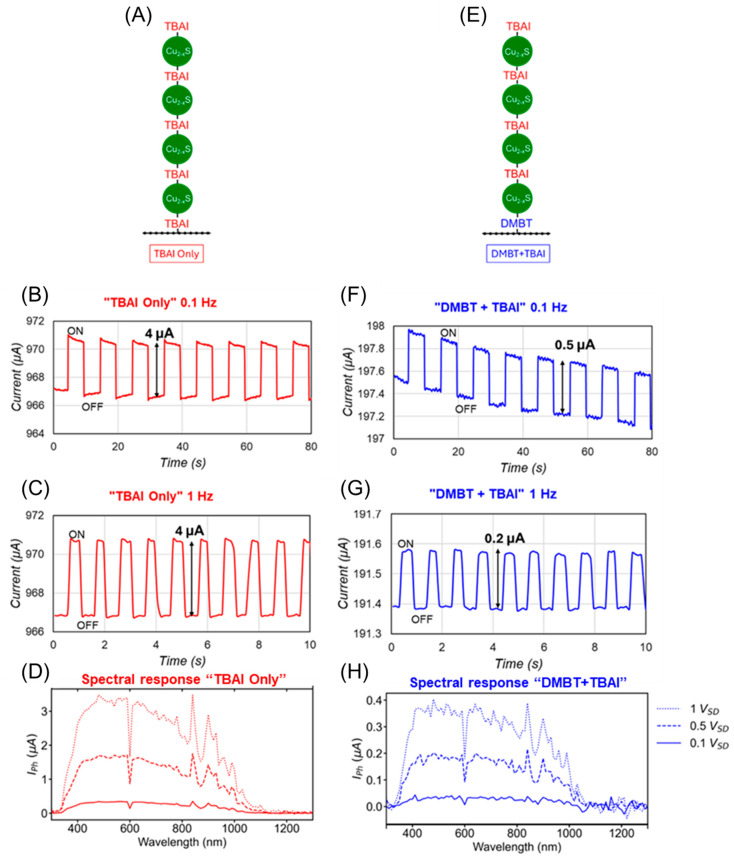
(**A**) TBAI-only photodetector based on four spin-coated layers of Cu_2−x_S NCs on SLG functionalised with TBAI molecules. (**B**,**C**) Photoresponse of the TBAI-only device under 0.1 Hz and 1 Hz light excitation, respectively. (**D**) Spectral response of TBAI-only devices showing strong photoresponse in the 400–1000 nm range for 0.1, 0.5 and 1 V_DS_. (**E**) DMBT + TBAI device is based also on four spin-coated layers of NCs but uses DMBT as a ligand for the first NC layer to improve coupling between NCs and SLG. (**F**,**G**) Photoresponse of DMBT + TBAI devices under 0.1 and 1 Hz light excitation. (**H**) DMBT + TBAI device showing strong photoresponse in the 400–1000 nm range for 0.1, 0.5 and 1 V_DS_.

The strong interaction between the NCs and SLG mediated by ligands at their interface prompted us to investigate the photocurrents in SLG-NCs photodetectors, comparing the effect of DMBT and TBAI ligands on the device performance. We prepared two types of hybrid photodetectors by spin-coating three layers of Cu_2_₋_x_S NCs on the SLG/Cu_2_₋_x_S/TBAI (“TBAI-only” device) and the SLG/Cu_2_₋_x_S/DMBT (“DMBT + TBAI” device) heterojunction, respectively, applying the TBAI treatment after the deposition of each of the three layers, in both cases (Figure 5A,E). In the first device, only TBAI was used due to its known effectiveness in coupling sulphur-based NCs. For the second device (Figure 5E), DMBT treatment was applied only to the first NC layer to leverage the stronger interaction it induces between Cu_2_₋_x_S NCs and SLG (Figure 4D). TBAI was used for the remaining layers to facilitate charge transfer between the NCs, taking advantage of its established effectiveness with sulphur-based materials [19].

The photoresponse of the heterojunction was measured under ON/OFF light stimulation (λ = 450 nm, 100 μW, Vbias = 0.5 V). For the TBAI-only device (Figure 5A) a clear increase in current is observed when light is turned ON (Figure 5B), resulting from the transfer of the photogenerated holes to p-type SLG, which increases the majority carrier density. The devices exhibit a photocurrent of 4 μA, a responsivity of 40 mA/W, a response time < 1 s, and a stable dark current under a periodic light excitation at 0.1 Hz. The fast response led us to test the photoresponse at 1 Hz (Figure 5C), where the ON-OFF states are clearly resolved, and the photocurrent remains at 4 μA. The specific detectivity for these “TBAI only” devices is 1.15 × 10^5^ Jones. Figure 5D shows the spectral response of the devices, showing a strong photocurrent in the 400–1000 nm range for 0.1, 0.5, and 1 V_DS_. In contrast, the DMBT + TBAI devices (Figure 5E) show a lower photoresponse with a photocurrent of 0.5 μA and a responsivity of 6 mA/W under a 0.1 Hz excitation, along with some drift in the dark current (Figure 5F). The specific detectivity for the “TBAI + DMBT” devices is lower at 1.72 × 10^4^ Jones, as expected. This reduction in responsivity and detectivity may be associated with the drop in mobility, which is linearly related to the photogain of the devices. The mobility of the TBAI devices is 4200 cm^2^/Vs, nearly 10-folds larger than for DMBT treatment of 530 cm^2^/Vs. This ratio is very similar to the drop in responsivity of 40 mA/W to 6 mA/W for the “DMBT-only” devices. Despite the reduced performance, the DMBT + TBAI devices still exhibit fast response times and can operate at faster speeds of 1 Hz (Figure 5G), though with a lower photocurrent (0.2 μA) and a responsivity of 2.4 μA. Figure 5H shows the spectral response for “DMBT + TBAI” devices also with a strong photocurrent in the 400–1000 nm range.

The photoresponse observed for the hybrid devices under light illumination can be attributed to the separation of the excitons photogenerated in the NCs. In the case of the TBAI-only devices, the built-in potential drives photogenerated holes from the NCs to SLG, increasing the majority carrier density, and thus enhancing the positive photocurrent, since SLG is p-doped in the presence of NCs with TBAI (Figure 4F) [3,28]. Meanwhile, the photoelectrons remain in the NCs, contributing to the photogating effect [3]. For the DMBT + TBAI devices, the NCs-SLG heterojunction facilitates the transfer of the photogenerated holes from the NCs to SLG, aided by the delocalisation through the aromatic π electron system of DMBT. This leads to a stronger Fermi level shift and a higher built-in potential [30]. However, the reduced mobility in the DMBT + TBAI devices results in an overall lower photoresponse compared to the TBAI-only devices.

Compared to previous hybrid devices using Cu_2_S−graphene heterojunctions, our devices exhibit significantly higher responsivity. For example, Su et al. [30] achieved a photocurrent of 3 nA under 30 mW illumination with a 10 V bias, achieved using reduced graphene oxide and Cu_2_S NCs prepared via hot injection, corresponding to a responsivity of 100–0.1 μA/W at 10 V bias voltage. In contrast, our TBAI-only devices demonstrate a photoresponsivity of 40 mA/W, while the DMBT + TBAI devices achieve 6 mA/W, both at a significantly lower bias of just 0.5 V. Similarly, graphene oxide decorated with CuS nanoparticles exhibit strong photoresponse of 20 mA/W but requires a much higher V_DS_ of 30 V and shows slower response times > 1 s [31]. These results underscore the superior performance of our devices, showcasing a remarkable enhancement in photocurrent due to the improved coupling between Cu_2−x_S NCs and SLG, enabled by TBAI and DMBT ligand treatments. Table 1 provides a detailed comparison of our devices with the aforementioned photodetectors based on graphene and CuS nanoparticles.

## 3. Materials and Methods

Materials. Copper(I) chloride (CuCl, 99.99%), sulphur powder (S_8_, 99.99%), 1-octadecene (ODE, technical grade 90%), oleic acid (OA, technical grade 90%), oleylamine (Olam, 70%), chloroform (CHCl_3_), ethanol (EtOH), tetrachloroethylene (TCE), 3,4-dimethylbenzenethiol (DMBT, 98%) and tetrabutylammonium iodide (TBAI), were purchased from Sigma-Aldrich. Single-layer graphene (SLG) grown by Chemical Vapor Deposition (CVD) was purchased from Graphenea.

Synthesis of Olam/OA-capped Cu_2−x_S NCs. The Cu_2−x_S NCs were synthesised by the hot injection method under air-free conditions by using a standard Schlenk line setup [20]. Solutions of S_8_ and CuCl_2_, selected as Cu and S precursors, respectively, were prepared in two separate three-necked flasks by dispersing in one flask, 0.5 mmol of CuCl_2_ (67 mg) in a mixed coordinating solvent mixture based on 1.5 mL (2.5 mmol) of OA and 3.5 mL (5 mmol) of Olam into 7.5 mL (10 mmol) of ODE, and in the second flask, 1 mmol of S_8_ powder (0.2 M) in 2.5 mL of OA and 2.5 mL of ODE. The two flasks were both heated up to 100 °C for 1 h under vacuum and vigorous magnetic stirring to remove air and moisture, and then their temperature was increased up to 150 °C. The S precursor reaction mixture was then cooled down to room temperature and swiftly injected into the copper precursor reaction flask heated up to 200 °C, followed by a temperature decrease down to 180 °C to allow NCs growth. The reaction mixture was left to react for 10 min, then cooled down to room temperature and purified by centrifugation at 950 rpm for 10 min, followed by re-dispersion in EtOH (Centric 322 A, Tehtnica). The collected precipitate was redispersed in 2.5 mL of TCE for further investigation.

Graphene transfer onto silicon: SLG was transferred onto a silicon (Si) chip (1 × 1 cm^2^) substrate 525 μm thick with a top 300 nm thick thermal silicon oxide (SiO_2_) layer. The transfer was performed using PMMA as a top supporting layer. The PMMA/graphene layer on copper was left floating on a 0.1 M sodium persulfate solution until copper was dissolved. Then the PMMA/graphene layer was transferred to two subsequent DI water baths and finally to a Si/SiO_2_ substrate and left to dry. Finally, PMMA is removed by acetone and isopropanol.

Deposition of Olam/OA-capped Cu_2−x_S NCs onto SLG. Two SLG/Cu_2−x_S heterojunctions were fabricated by spin coating (2500 rpm for 10 s) 50 µL of a 25.7 μM Olam/OA-Cu_2−x_S NCs solution in TCE onto SLG. Then, to single out the effects of the different molecules at the SLG/NC interface, two heterojunctions were treated separately by spin-coating for 30 s, 0.03 M methanol solutions of TBAI and DMBT, respectively. This process replaced the Olam/OA capping layer on the NC surface with TBAI and DMBT. After treatment, the heterojunctions were rinsed with methanol using spin-coating at 2500 rpm for 10 s. Subsequently, Cu_2−x_S NCs-Olam/OA were deposited on both heterojunctions by spin-coating 50 µL of a 25.7 μM Cu_2_₋_x_S NC-Olam/OA solution in TCE, followed by treatment with a 0.03 M TBAI methanol solution, and finally rinsed with methanol using spin-coating at 2500 rpm for 10 s.

### Characterisation

Transmission Electron Microscopy (TEM). TEM analyses were performed by a JEOL JEM1011 electronic microscope (Jeol, Tokyo, Japan) operating at 100 kV, equipped with a high-resolution CCD camera. Carbon-coated copper grids were dipped in NC solutions in TCE, then the solvent was left to evaporate.

UV-Vis-NIR Spectroscopy. UV-Vis-NIR absorption spectra of Cu_2−x_S NCs solutions were collected by a 1 cm path length quartz cuvettes using a Cary Varian 5000 UV/Vis/NIR spectrophotometer (Agilent Technologies, Santa Clara, CA, USA) equipped with an integration sphere to measure the diffuse reflectance.

Fourier Transform Infrared-Attenuated Total Reflectance (FTIR-ATR) Spectroscopy**.** The FTIR characterisation was carried out by a Varian 670 FTIR spectrometer (Varian, Palo Alto, CA, USA) equipped with a diamond attenuated total reflection (ATR) accessory of 2 mm and a deuterated triglycine sulfate detector. 1 µL of each sample was put on the internal reflection element, and then the spectrum was recorded in the range of 4000–400 cm^−1^ using 16 accumulation scans acquired with a nominal resolution of 1 cm^−1^.

Raman investigation. Raman spectra were collected by a LabRAM HR Horiba-Jobin Yvon spectrometer (Horiba Ltd., Kyoto, Japan) with a 532 nm excitation laser source. Measurements were carried out under ambient conditions at low laser power (1 mW) to avoid laser-induced damage. The Raman band recorded from a silicon wafer at 520 cm^−1^ was used to calibrate the spectrometer, and the accuracy of the spectral measurement was 1 cm^−1^.

Optoelectronic characterisation: The light intensity was measured with a standard silicon photodiode power sensor (S120VC, Thorlabs, Newton, NJ, USA) at the same position as the samples. Current-voltage data and transconductance measurements were measured using a Keithley 2400 source meter (Tektronix, Beaverton, OR, USA) under a 450 nm laser diode (CPS450, Thorlabs, USA).

## 4. Conclusions

Hybrid photodetectors integrating heterojunctions of a single layer graphene (SLG) and oleylamine and oleic acid (Olam/OA)-capped Cu_2−x_S NCs (SLG/Cu_2−x_S) have been fabricated. The organic coated NCs have been spin-coated onto SLG and then treated with 3,4-dimethylbenzenethiol (DMBT) and tetrabutylammonium iodide (TBAI) to replace the long alkyl chain ligands. Raman spectroscopy and transconductance measurements have demonstrated charge coupling interactions between the NCs and SLG in the SLG/Cu_2−x_S/DMBT heterojunction, which are stronger than in the TBAI counterpart. These enhanced interactions are attributed to the π-π coupling between SLG and DMBT, facilitating electron transfers from the NCs to SLG under thermal equilibrium conditions. However, the stronger coupling also reduces carrier mobility, leading to lower overall responsivity compared to TBAI-only devices, which shows a photocurrent of 4 μA, with high responsivity (40 mA/W) and fast response times (<1 s). Notably, the photoresponsivity of our hybrid photodetectors exceeds that of state-of-the-art photodetector devices based on similar hybrid nanocomposites. This marks a significant advancement towards the development of low-cost, environmentally friendly photodetectors that avoid the use of hazardous heavy metals.

## Figures and Tables

**Table 1:** Performance metrics of photodetectors based on graphene and CuS nanomaterials.

Material	Bias Voltage V	Wavelength nm	Responsivity mA/W	Frequency Operation Response Time	Ref.
Reduced Graphene Oxide Cu_2_S	10	405	0.04–0.0004	0.02 Hz	[30]
Graphene Oxide- CuS	30	480	24.65	0.05 Hz 2 s	[31]
CVD Graphene Cu_2−x_S	0.5	450	40	1 Hz <1 s	This work

## Data Availability

Data are contained within the article.
